# Trigonelline Attenuated Sepsis-Induced Acute Kidney Injury by Activating NAD^+^/SIRT1 Pathway

**DOI:** 10.33549/physiolres.935486

**Published:** 2025-06-01

**Authors:** Wenming LV, Dan CAO, Fan YANG

**Affiliations:** 1 Department of Anesthesiology & Operation, Suining Central Hospital, Suining, China; 2 Basic Laboratory, Suining Central Hospital, Suining, China; 3 Department of Anesthesiology & Operation, Sichuan Provincial People’s Hospital East Sichuan Hospital & Dazhou First People’s Hospital, Dazhou, China

**Keywords:** Trigonelline, Sepsis-induced acute kidney injury, NAD^+^, SIRT1

## Abstract

Sepsis-induced acute kidney injury (SAKI) is one of the most frequent complications in patients with sepsis and is strongly associated with poor clinical outcomes. Trigonelline (TRL), a bioactive pyridine alkaloid isolated from fenugreek, has exhibited therapeutic effects on various diseases. This study aimed to investigate the effects of TRL on SAKI and whether TRL exerted its function *via* NAD^+^/SIRT1 pathway activation. A single dose (10 mg/kg body weight) of lipopolysaccharide (LPS) was intraperitoneally administered to establish a mouse SAKI model. After 24 h, compared with the control group, the plasma levels of kidney function indicators creatinine and blood urea nitrogen, oxidative stress indicators hydrogen peroxide and malondialdehyde, and inflammatory factors tumor necrosis factor-α and interleukin-1β were significantly increased. Meanwhile, hematoxylin and eosin staining results revealed that LPS treatment caused glomerular structure disruption, renal tubular luminal narrowing, and renal tubular structure deterioration. TRL treatment significantly reduced the plasma kidney function indicators, oxidative stress, and inflammatory factors levels in the SAKI mice, accompanied by improvements in the renal pathological changes. Furthermore, TRL treatment increased the NAD^+^ levels, upregulated the SIRT1 expression, and downregulated the NOX4 expression in the kidney of the SAKI mice. Subsequently, EX-527, a selective SIRT1 inhibitor, was used for inhibiting SIRT1, and it reversed the protective effect of TRL in SAKI. Our results revealed that TRL improved renal function and alleviated inflammation and oxidative stress in SAKI mice by NAD^+^/SIRT1 pathway activation. Therefore, TRL may be a potential therapeutic approach for SAKI treatment.

## Introduction

Sepsis, a systemic inflammatory response syndrome, is caused by a dysregulated host response to infection, which can induce severe multiorgan dysfunction when not effectively controlled [1]. As the kidney is one of the initial organs affected during sepsis, sepsis-induced acute kidney injury (SAKI) is one of the most frequent complications in patients with sepsis and is strongly associated with poor clinical outcomes, including longer hospital stays, a higher risk of mortality, and the development of chronic comorbidities [2]. However, the therapeutic approach for this condition is frequently limited to symptomatic treatments. Several mechanisms have been proposed to play a significant role in the pathogenesis of SAKI, including excessive inflammation, oxidative stress injury, mitochondrial dysfunction, autophagy, and apoptosis [3–5]. Sirtuin, a class III histone deacetylase, is a nicotinamide adenosine dinucleotide (NAD^+^)-dependent deacetylase. Sirtuins remove acetyl groups from several target proteins using NAD^+^ as a cosubstrate to participate in various biological processes. Sirtuin-1 (SIRT1), the most widely expressed and well-investigated member of the sirtuin family of proteins, contributes to maintaining homeostasis by regulating the inflammatory response, inhibiting oxidative stress, improving mitochondrial function, and reducing autophagy and apoptosis [6–8]. In contrast, SIRT1 deficiency or reduced activity is strongly involved in the pathogenesis of various diseases, including sepsis and SAKI. SIRT1 has been reported to attenuate SAKI by promoting autophagy mediated by Beclin1 deacetylation [9]. Additionally, resveratrol, the SIRT1 activator, can prevent sepsis and its related complications in several ways [10]. Therefore, SIRT1 is a promising target in the treatment of SAKI.

Trigonelline (TRL), a bioactive pyridine alkaloid, is the main pharmacological ingredient of the traditional Chinese herb fenugreek and is also noted in coffee [11]. TRL has been demonstrated to possess therapeutic effects on diabetes, central nervous system diseases, and cancers through its biological activities, including hypolipidemic, memory-improving, neuroprotective, antioxidant, antidia-betic, and anticancer effects [12–13]. TRL has been recently reported to act against renal fibrosis in the process of diabetic kidney disease by targeting Smad7 [14]. Furthermore, reduced TRL excretion has been associated with the disruption of the NAD^+^ and SIRT1 pathways [15]. Moreover, coffee consumption has been shown to mitigate the risk of AKI [16]. However, the effects of TRL on SAKI and the underlying molecular mechanisms have not been explored.

This study aimed to investigate the effects of TRL on SAKI and whether TRL exerted its function *via* NAD^+^/SIRT1 pathway activation.

## Methods

### Animals and treatments

Male C57BL/6J mice (8–10 weeks old) were obtained from Chengdu Gembio Co., Ltd. (Chengdu, China) and housed under standard conditions (12-h light/dark cycle) under a constant temperature of 22–24 °C and humidity of 60 %. All animal experiments complied with the relevant regulations of the Animal Ethics Committee of Suining Central Hospital. Experiments were performed according to the Guidelines for the Care and Use of Laboratory Animals of the US National Institutes of Health. Following acclimatization, 24 mice were randomly divided into the following four groups (n=6 per group): Control, lipopolysaccharide (LPS), LPS + TRL, and LPS + TRL + EX-527 groups. The mice in the LPS, LPS + TRL, and LPS + TRL + EX-527 groups were intraperitoneally injected with a single dose (10 mg/kg body weight) of LPS to establish a mouse SAKI model, and the Control group was intraperitoneally administered with the equivalent volume of saline. Before LPS treatment, the mice in the LPS + TRL and LPS + TRL + EX-527 groups were pretreated with TRL (50 mg/kg/day, dissolved in ddH_2_O) *via* gavages for 7 days. Simultaneously, the mice in the LPS + TRL + EX-527 group were intraperitoneally injected with the SIRT1 inhibitor EX-527 (5 mg/kg, dissolved in DMSO) for 7 days. LPS, TRL, and EX-527 were purchased from Solarbio Science & Technology Co., Ltd. (Beijing, China).

Following a 24-h LPS treatment, the mice were sacrificed, and blood and kidney tissue samples of the mice were collected. Plasma was prepared by centrifuging the blood samples at 3000 rpm for 15 min at 4 °C and frozen at −80 °C for subsequent experimental analysis. The right kidney tissue samples were fixed with 4 % paraformaldehyde, and the left kidney tissue samples were frozen at −80 °C.

### Plasma biochemical indicators of kidney function analysis

The plasma levels of the kidney function indicators, creatinine (CRE) and blood urea nitrogen (BUN), were measured using biochemical kits following the manufacturer’s instructions: the CRE detecting kits were purchased from Nanjing Jiancheng Bioengineering Institute (Nanjing, China), and the BUN detecting kits were purchased from Solarbio Science & Technology Co., Ltd. (Beijing, China).

### Plasma oxidative stress and inflammatory cytokines analysis

The plasma levels of the oxidative stress indicators, hydrogen peroxide (H_2_O_2_) and malondialdehyde (MDA), were determined using the corresponding assay kits (Solarbio Science & Technology Co., Ltd., Beijing, China) following the manufacturer’s instructions. The plasma inflammatory cytokines, tumor necrosis factor (TNF)-α and interleukin (IL)-1β, were measured using enzyme-linked immunosorbent assay kits (Proteintech, USA) following the manufacturer’s instructions.

### Measurement of kidney NAD^+^ concentration

The kidney tissue samples were weighed and homogenized with NAD^+^/NADH extract buffer. Following centrifugation, the supernatant was used for measuring NAD^+^ concentration using the NAD^+^/NADH assay kit (Beyotime Biotechnology, Shanghai, China) following the manufacturer’s instructions.

### Histomorphological assay

After fixing with 4 % formaldehyde, the kidney tissue samples were embedded in paraffin. The samples were sliced into 4-μm sections and stained with hematoxylin and eosin (HE) reagent. Subsequently, the renal morphology was observed under a microscope. Two researchers, who were blinded to the experiment, scored the kidney injury in five randomly selected fields from each section based on the percentage of cortical tubular damage as follows: 0, no damage; 1, damage <25 % of the tubular area; 2, damage between 26 % and 50 % of the tubular area; 3, damage between 51 % and 75 % of the tubular area; and 4, damage between 76 % and 100 % of the tubular area.

### Western blot analysis

The kidney tissue samples used for Western blotting were lysed in RIPA buffer (Beyotime Biotechnology, Shanghai, China) containing phosphatase and protease inhibitor cocktail (Roche, Merck, Germany) and quantified using BCA reagent. Equal amounts of protein were separated by 10 % sodium dodecyl sulfate-polyacrylamide gel electrophoresis and transferred onto polyvinylidene fluoride membranes (Millipore, Billerica, MA, USA). Subsequently, the membranes were blocked in 5 % BSA for 1 h and incubated with the following primary antibodies: SIRT1 (1:1000; Proteintech, USA) and NADPH Oxidase-4 (NOX4, 1:1000; Proteintech, USA) at 4 °C overnight, and GAPDH (1:1000; Proteintech, USA) was used as the loading control. Following incubation with the secondary antibodies for 1 h, the target protein bands were visualized using the ECL exposure system and quantified using ImageJ (National Institutes of Health, Bethesda, MD, USA).

### Statistical analyses

All experimental data were presented as means ± SEM, and statistical analysis was performed using Statistical Package for the Social Sciences version 21 (SPSS Inc., Chicago, IL, USA). Independent *t*-test was used to compare values between the two groups, and the differences among the three groups were assessed using one-way analysis of variance, followed by the least significant difference test. P<0.05 was considered statistically significant.

## Results

### Trigonelline protected against LPS-induced SAKI in mice by NAD^+^/SIRT1 pathway activation

**Fig. 1:**
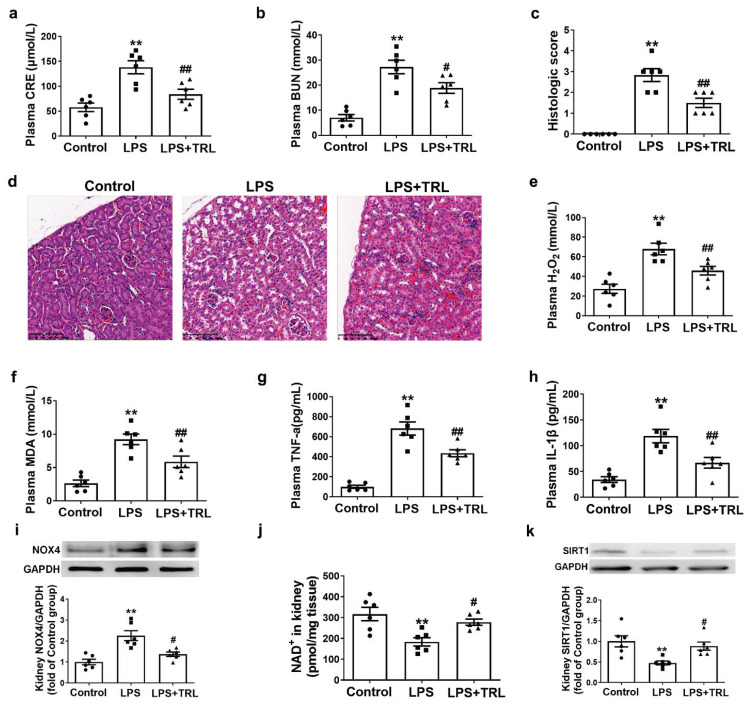
Trigonelline protected against LPS-induced SAKI in mice by activating NAD^+^/SIRT1 pathway: (**A**) Plasma creatinine (CRE) levels. (**B**) Plasma blood urea nitrogen (BUN) levels. (**C–D**) Representative and quantitative analysis for HE-stained kidney tissues (scale bar = 100 μm). (**E**) Plasma hydrogen peroxide (H_2_O_2_) levels. (**F**) Plasma malondialdehyde (MDA) levels. (**G**) Plasma TNF-α levels. (**H**) Plasma IL-1β levels. (**I**) Representative Western blots and quantitative analysis for NOX4 protein expression in kidney tissues. (**J**) The NAD^+^ levels in kidney tissues. (**K**) Representative Western blots and quantitative analysis for SIRT1 expression. Results are means ± SEM. * P<0.05 vs. Control group; ** P<0.01 vs. Control group; ^#^ P<0.05 vs. LPS group; ^##^ P<0.01 vs. LPS group; P<0.05 was considered significant.

The SAKI mouse model was established by injecting mice with LPS to induce sepsis. Following the 24-h LPS treatment, the LPS group showed significantly increased levels of kidney function indicators, plasma CRE and BUN, compared with the Control group (Fig. 1A–B). TRL treatment significantly decreased the plasma CRE and BUN levels in the LPS-induced SAKI mice. Morphologically, HE staining results revealed that LPS treatment resulted in glomerular structure disruption, renal tubular luminal narrowing, and renal tubular structure deterioration. TRL treatment alleviated these pathological lesions in the LPS-induced SAKI mice (Fig. 1C–D). SAKI was associated with oxidative stress injury and inflammation. As shown in Figures 1E–F, the LPS group exhibits significantly increased levels of oxidative stress indicators, plasma H_2_O_2_ and MDA, compared with the Control group, accompanied by an increase in the plasma levels of inflammatory factors, including TNF-α and IL-1β (Fig. 1G–H). TRL treatment markedly attenuated the plasma H_2_O_2_, MDA, TNF-α, and IL-1β levels in the LPS-induced SAKI mice. The Western blot analysis results revealed that NOX4 protein expression was upregulated in the kidneys of LPS-induced SAKI mice, which were downregulated by TRL treatment (Fig. 1I). As shown in Figure 1J, the LPS group exhibits decreased NAD^+^ levels in the kidney compared with the Control group, and SIRT1 protein expression was also downregulated in the kidney (Fig. 1K). TRL treatment increased the NAD^+^ levels in the kidney and upregulated the SIRT1 protein expressions, indicating NAD^+^/SIRT1 pathway activation.

### EX-527 blocked the protective effect of trigonelline in LPS-induced SAKI

**Fig. 2:**
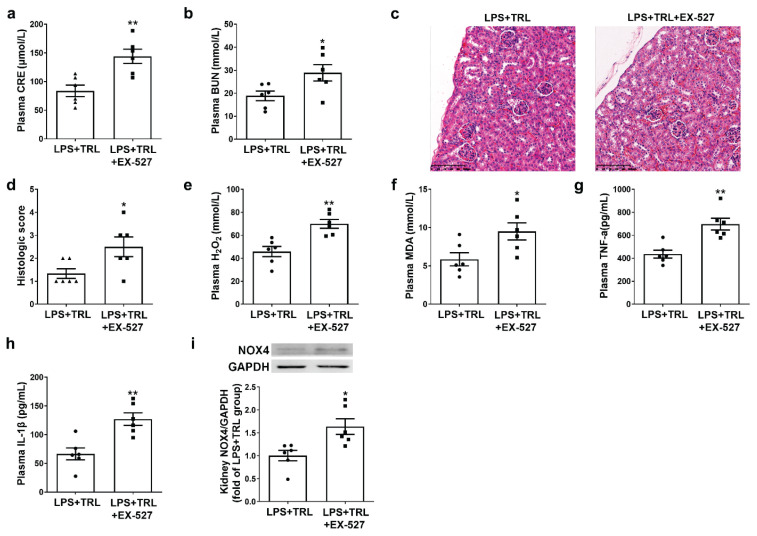
EX-527 blocked the protective effect of trigonelline in LPS-induced SAKI: (**A**) Plasma creatinine (CRE) levels. (**B**) Plasma blood urea nitrogen (BUN) levels. (**C–D**) Representative and quantitative analysis for HE-stained kidney tissues (scale bar = 100 μm). (**E**) Plasma hydrogen peroxide (H_2_O_2_) levels. (**F**) Plasma malondialdehyde (MDA) levels. (**G**) Plasma TNF-α levels. (**H**) Plasma IL-1β levels. (**I**) Representative Western blots and quantitative analysis for NOX4 protein expression in kidney tissues. Results are means ± SEM. * P<0.05 vs. LPS + TRL group; ** P<0.01 vs. LPS + TRL group; P<0.05 was considered significant.

The LPS + TRL + EX-527 group showed significantly increased plasma CRE and BUN levels compared with the LPS + TRL group (Fig. 2A–B), accompanied by deterioration of the renal morphology (Fig. 2C–D). Moreover, compared with the LPS + TRL group, the plasma H_2_O_2_, MDA, TNF-α, and IL-1β levels were significantly increased following EX-527 treatment (Fig. 2E–H). Additionally, kidney NOX4 protein expression was increased following EX-527 treatment (Fig. 2I).

## Discussion

We here investigated the therapeutic effects of TRL on SAKI and explored the underlying molecular mechanisms. Our results demonstrated that TRL improved renal function and alleviated inflammation and oxidative stress in SAKI mice by activating the NAD^+^/SIRT1 pathway.

SAKI is a frequent and life-threatening complication of sepsis caused by a dysregulated host response to an infection, which is associated with poor clinical outcomes and high mortality. Despite this, effective clinical treatment for SAKI remains elusive. LPS, a major component of the cell membrane in gram-negative bacteria, is a well-characterized pathogen-associated molecular pattern and the main inducing factor of SAKI in patients with critical illness. Therefore, LPS-induced SAKI is a widely accepted and reliable model used for investigating the pathophysiological mechanisms and therapeutic effects in preclinical research [17–18]. In the present study, the intraperitoneal injection of LPS was also employed for establishing SAKI mice models. Following the 24-h LPS treatment, the plasma levels of kidney function indicators, CRE and BUN levels, were significantly increased compared with those in the control group. Meanwhile, following LPS treatment, HE staining results showed glomerular structure disruption, renal tubular luminal narrowing, and renal tubular structure deterioration. The abovementioned results indicate that LPS treatment induces SAKI, which agrees with the results of several studies [19–20]. Subsequently, we investigated the protective role of TRL on SAKI. TRL, a pyridine alkaloid contained in some plants, is also a vitamin derivative, which is beneficial for humans against various diseases. In the present study, 50 mg/kg/day of TRL was administered *via* gavages for 7 days. This dose was much lower than its lethal dose of 5000 mg/kg [21] and did not induce any adverse effects [22]. TRL treatment significantly reduced the plasma CRE and BUN levels in LPS-induced SAKI mice, accompanied by improvements in the renal pathological changes, indicating that TRL exhibits favorable renoprotective effects in LPS-induced SAKI mice. Consistent with our results, TRL has also been shown to ameliorate diabetic nephropathy [23] or prevent kidney stone formation [24].

Oxidative stress injury and inflammation play a pivotal role in the pathophysiology of SAKI [25–26]. Meanwhile, TRL has been demonstrated to exert antioxidative and anti-inflammatory effects. For example, TRL exerted antidepressant- and anxiolytic-like effects in a mouse model of maternal separation stress by reducing oxidative stress and increasing the antioxidant capacity [27]. Furthermore, by targeting NF-κB/NLRP3/IL-1β signaling, TRL mitigated bleomycin-induced pulmonary inflammation and fibrosis [28]. Consistent with previous studies, following LPS treatment, the plasma levels of oxidative stress indicators, H_2_O_2_ and MDA, were significantly increased, accompanied by an increase in the plasma levels of inflammatory factors, TNF-α and IL-1β, whereas TRL treatment markedly attenuated the plasma H_2_O_2_, MDA, TNF-α, and IL-1β levels in LPS-induced SAKI mice. Moreover, Khalili *et al*. [29] reported that TRL mitigated LPS-induced learning and memory impairment in a rat model owing to its antioxidative and anti-inflammatory properties.

NOX4, as the major source of reactive oxygen species, was upregulated in sepsis to induce oxidative stress and inflammation [30]. Some pieces of evidence have recently revealed that SAKI is mediated by NOX4 [31], and NOX4 deletion or its pharmacological inhibition by GKT137831 can alleviate LPS-injured renal function and pathology in mice [32]. In the present study, our results showed that NOX4 protein expression was upregulated in the kidneys of LPS-induced SAKI mice, which were downregulated by TRL treatment. Consistent with our findings, TRL ameliorated oxidative stress in type 2 diabetic Goto-Kakizaki rats by downregulating the gene expressions involved with NADPH oxidase and the mitochondrial electron transfer system [33]. A growing body of evidence supports the critical role of SIRT1, the highly conserved NAD^+^-dependent deacetylase, in the development of the sepsis process [34–35]. The activated SIRT1 was competitively bound to p22 phox, inhibiting NOX4 activation and facilitating NOX4 ubiquitination and degradation to reduce oxidative stress and inflammation [36]. The loss of SIRT1 could enhance NOX4 expression [37], whereas chronic SIRT1 treatment resulted in reduced NOX4 expression [38]. In a most recent study, TRL was noted to be an NAD^+^ precursor that improved muscle function during aging [39]. Consistent with this study, we observed that the NAD^+^ levels in the kidney were decreased following LPS treatment, and SIRT1 protein expression was also downregulated in the kidney. TRL treatment increased the NAD^+^ levels and upregulated the SIRT1 protein expression in the kidney. Meanwhile, the treatment of SIRT1 inhibitor EX-527 blocked the protective effect of TRL in LPS-induced SAKI, indicating that the NAD^+^/SIRT1 pathway was activated following TRL treatment. Ex-527 could inhibit SIRT1 by exploiting the unique NAD^+^-dependent deacetylation mechanism [40]. Consistent with the results of our study, Wang *et al*. [41] reported that EX-527 counteracted the protective effect of β-nicotinamide mononucleotide (NMN) in septic mice by blocking the SIRT1 pathway. Furthermore, SIRT1 inhibition by EX-527 reversed the effects of NMN-induced M2 macrophage polarization sepsis-induced acute lung injury [42]. Additionally, EX-527 partially abolished the antioxidant and anti-apoptotic effects of polysulfide in diabetic nephropathy [43].

## Conclusions

In conclusion, our results revealed that TRL improved renal function and alleviated inflammation and oxidative stress in SAKI mice by activating the NAD^+^/SIRT1 pathway. Therefore, TRL may be a potential therapeutic approach for future SAKI treatment.
